# Amelioration of Obesity-Related Disorders in High-Fat Diet-Fed Mice following Fecal Microbiota Transplantation from Inulin-Dosed Mice

**DOI:** 10.3390/molecules28103997

**Published:** 2023-05-10

**Authors:** Yinli Huang, Na Ying, Qihui Zhao, Junli Chen, Sin-Yeang Teow, Wei Dong, Minjie Lin, Lingling Jiang, Hong Zheng

**Affiliations:** 1 Department of Endocrinology, Pingyang Affiliated Hospital of Wenzhou Medical University, Wenzhou 325400, China; yinli0423@126.com (Y.H.); a281532331@gmail.com (W.D.); huahuaqbz@163.com (M.L.); 2 School of Pharmaceutical Sciences, Wenzhou Medical University, Wenzhou 325035, China; yingna0219@163.com (N.Y.); zhaoqihui0614@163.com (Q.Z.); chjunli123@163.com (J.C.); 3 College of Science and Technology, Wenzhou-Kean University, Wenzhou 325060, China; 4 Wenzhou Municipal Key Laboratory for Applied Biomedical and Biopharmaceutical Informatics, Wenzhou-Kean University, Wenzhou 325060, China

**Keywords:** obesity, inulin, gut microbiota, inflammation, liver

## Abstract

The role of inulin in alleviating obesity-related disorders has been documented; yet, its underlying mechanisms still need to be further investigated. This study attempted to elucidate the causative link between the gut microbiota and the beneficial effect of inulin on obesity-related disorders via transferring the fecal microbiota from inulin-dosed mice to high-fat diet (HFD)-induced obese recipient mice. The results show that inulin supplementation can decrease body weight, fat accumulation, and systemic inflammation and can also enhance glucose metabolism in HFD-induced obese mice. Treatment with inulin reshaped the structure and composition of the gut microbiota in HFD-induced obese mice, as characterized by increases in the relative abundances of *Bifidobacterium* and *Muribaculum* and decreases in *unidentified_Lachnospiraceae* and *Lachnoclostridium*. In addition, we found that these favorable effects of inulin could be partially transferable by fecal microbiota transplantation, and *Bifidobacterium* and *Muribaculum* might be the key bacterial genera. Therefore, our results suggest that inulin ameliorates obesity-related disorders by targeting the gut microbiota.

## 1. Introduction

According to the Atlas report, obesity has emerged as a widespread global crisis and is projected to affect one billion people in the world by 2030 [1]. Obesity represents a significant threat as it increases the likelihood of a range of non-communicable diseases, such as diabetes, cardiovascular disease, hypertension, and cancers [2]. Given the significant impact of obesity on public health, its prevention and treatment are of the utmost importance but continue to pose a major challenge [3,4].

Thegut microbiotahas been regarded as a key mediator for the onset and progression of obesity and its associated disorders [5,6]. Several possible mechanisms underlying the impact of the gut microbiota on obesity and its associated disorders are proposed, such as immunity [7], inflammation [8], energy homeostasis [9], gut hormones [10], bile acid metabolism [11], and gut permeability [12]. Thus, modulating gut microbiota to treat obesity represents a valuable avenue [6,13]. Currently, plant-based diets are appreciated for their favorable effects on the gut flora and obesity [14,15]. Wang et al. reported that *Lyophyllumdecastes* polysaccharides alleviated high-fat diet (HFD)-driven obesity in mice via altering the gut microbiota and enhancing energy expenditure [16]. Previously, we also revealed that *Dendrobium officinale* dietary fiber can reshape the gut microbiome and host metabolism and reduce systemic inflammation and thereby prevent HFD-induced obesity in mice [17]. In addition to polysaccharides, other components from dietary plants also have a constructive role in the prevention and management of obesity, including polypeptides [18], polyphenols [19], resveratrol [20], nuciferine [21], kaempferol [22], and curcumin [23]. Hence, modification of the gut microbiota by plant-derived natural products serves as a potential approach to the treatment of obesity.

Inulin, a type ofoligosaccharide, is widely used as a prebiotic [24]. Inulin has also been reported to confer positive effects on the management of obesity-associated disorders, including adiposity [25], liver injury [26], inflammation [27], insulin resistance [28], intestinal barrier injury [29], and metabolic disorders [30]. In this study, HFD-fed mice were subjected to a 10-week intragastric administration of inulin, and the subsequent effects on the intestinal microbiome and obesity-related disorders were investigated. To further confirm the causal role of the gut microbiota in the protective effect of inulin against obesity-related disorders, fecal microbiota transplantation from inulin-dosed mice to HFD-induced obese recipient mice was performed. Our results not only provided more evidence on the anti-obesity effects of inulin, but also confirmed the gut microbiota as a crucial target of inulin.

## 2. Results

### 2.1. Inulin Alleviates Obesity-Related Disorders in HFD-Induced Obese Mice

**Figure 1:**
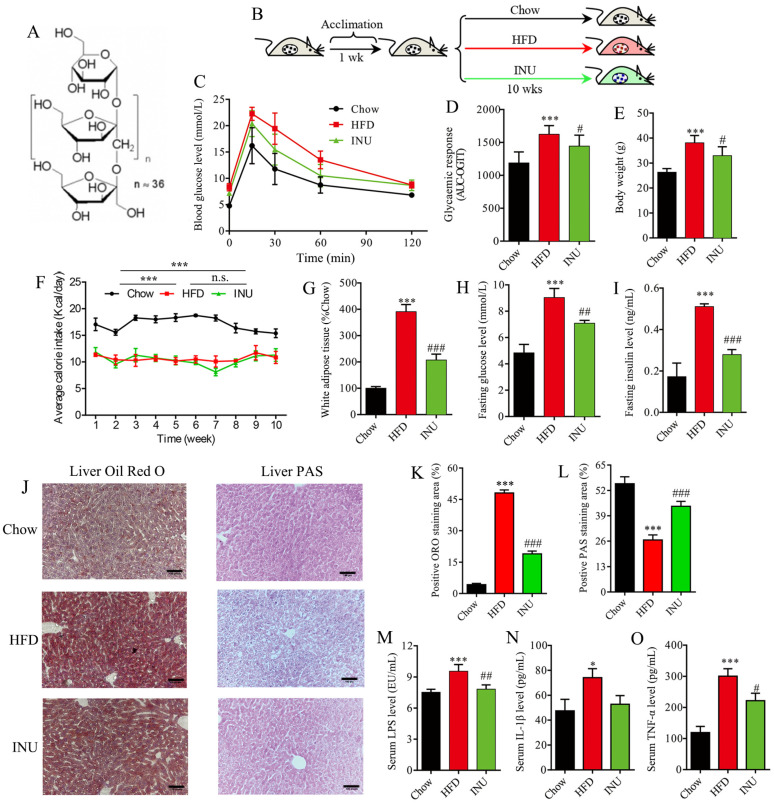
Inulin supplementation reduces obesity-related disorders in high-fat diet (HFD)-induced obese mice. (**A**) The structure of inulin; (**B**) inulin (INU) treatment: mice were fed with HFD and supplemented with daily intake of INU by gavage or normal chow (Chow) as a control group for 10 weeks; (**C**) oral glucose tolerance test (OGTT); (**D**) area under the curve (AUC) of the OGTT; (**E**) body weight; (**F**) daily calorie intake in each mouse (n = 3 cages; 3 mice per cage); (**G**) white adipose tissue weight; (**H**) fasting glucose level; (**I**) fasting insulin level; (**J**) histopathological examination: the liver sections were stained with oil red O (ORO) solution and periodic acid–Schiff (PAS) for evaluating lipid droplets and glycogen, respectively; scale bar =200 μm; (**K**) the percentage of positive ORO staining area; (**L**) the percentage of positive PAS staining area; (**M**) serum lipopolysaccharide (LPS) level; (**N**) serum IL-1β level; (**O**) serum TNF-α level. The statistical significance of the differences between two groups was evaluated by two-tailed unpaired Student’s *t*-test: * HFD vs. Chow; ^#^ INU vs. HFD. Significant level: * *p* < 0.05; *** *p* < 0.001; ^#^ *p* < 0.05; ^##^ *p* < 0.01; ^###^ *p* < 0.001.

To study the impact of inulin (INU, Figure 1A) intake on obesity-related disorders, mice were fed with HFD and simultaneously given INU by gavage, while normal mice chow (Chow) was givento a control group for 10 weeks (Figure 1B). Compared with the chow-fed mice, the OGTT experiments revealed that HFD feeding impaired glucose clearance ability in the mice; this ability could be improved after INU treatment (Figure 1C,D). Figure 1E shows that body weight was drastically increased in the mice after HFD compared to that of thechow-fed mice, although the HFD-fed mice had a lower daily calorie intake than the chow-fed mice (Figure 1F). This result may indicate that HFD resulted in obesity beyond the food calorie intake in mice. On the one hand, HFD can alter the gut microbiota to absorb more energy from food [31,32]. On the other hand, HFD has also been reported to cause endotoxemia and systemic inflammation, leading to obesity [33,34]. More notably, the body weight of the HFD-fed mice was significantly reduced after INU supplementation (Figure 1E), whichwas independent of the daily calorie intake (Figure 1F). The treatment with INU significantly decreased white adipose tissue (WAT) accumulation in the HFD-induced obese mice, as shown in Figure 1G. In addition, we observed that INU administration could significantly suppress HFD-driven increases in fasting glucose and insulin levels in the mice, as illustrated in Figure 1H,I, respectively. The histopathological results showed that HFD induced lipid droplet accumulation in the livers of themice but that this could be relieved after INU supplementation (Figure 1J,K). As glycogen synthesis is also beneficial in improving blood glucose homeostasis, liver PAS staining was examined in this study. We observed a significantly lower hepatic glycogen level in the HFD-induced obese mice compared with that in thechow-fed mice; however, its level could be markedly increased in the obese mice with the INU supplement (Figure 1J,L). The HFD-induced obese mice exhibited significantly increased levels of serum LPS (Figure 1M) and proinflammatory factors such as IL-1β (Figure 1N) and TNF-α (Figure 1O) relative to the chow-fed mice. Yet, notably, their levels could be significantly decreased in the HFD-induced obese mice after INU treatment (Figure 1M–O). Taken together, our results reveal that inulin can exert a beneficial role in the management of obesity and the associated disorders induced by HFD.

### 2.2. Inulin Alters the Composition of the Gut Microbiota in HFD-Induced Obese Mice

**Figure 2:**
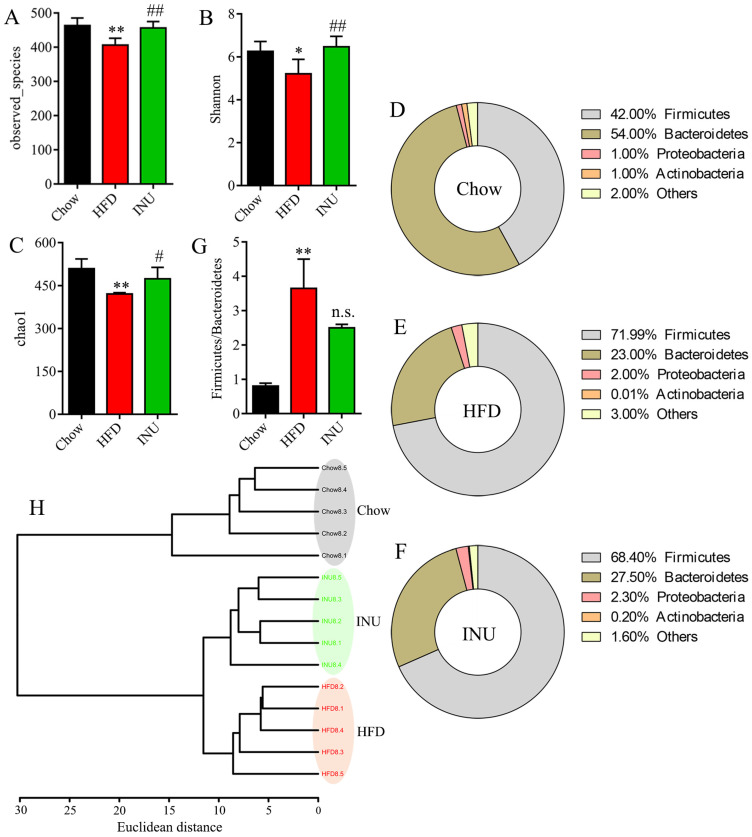
Inulin alters the gut microbiota composition in high-fat diet (HFD)-induced obese mice. (**A**) Observed species, (**B**) Shannon, and (**C**) chao1 indexes of the gut microbiota in mice receiving HFD (HFD), normal chow (Chow), and HFD supplemented with inulin (INU); (**B**–**D**) the percentages of the gut microbiota at the phylum level in the (**D**) Chow, (**E**) HFD, and (**F**) INU groups; (**G**) the ratio of *Firmicutes*/*Bacteroidetes*;(**H**) cluster analysis based on Ward’s method with Euclidean distance using the gut microbiome at the genus level. The significance of the differences between the two groups was evaluated by two-tailed unpaired Student’s *t*-test: * HFD vs. Chow; ^#^ INU vs. HFD. Significant level: * *p* < 0.05; ** *p* < 0.01; ^#^ *p* < 0.05; ^##^ *p* < 0.01; n.s., no significant difference.

To investigate the impact of INU on the gut microbiome, fecal samples were collected and analyzed by 16S rRNA sequencing. The gut microbial α-diversitywas significantly reduced in the HFD-induced obese mice compared with that of the chow-fed mice and notably increased after INU treatment, as shown in the observed species (Figure 2A), Shannon (Figure 2B) and chao1 (Figure 2C). At the phylum level, we found that HFD intake increased the percentages of *Firmicutes* and *Proteobacteria* but decreased the percentages of *Bacteroidetes* and *Actinobacteria* in the mice (Figure 2D,E). After INU supplementation, the structure of the gut microbiota was slightly reversed in the HFD-induced obese mice as indicated by lower *Firmicutes* and higher *Bacteroidetes* and *Actinobacteria* (Figure 2E,F). The ratio of *Firmicutes*/*Bacteroidetes* was significantly higher in the HFD-induced obese mice compared to that of the chow-fed mice, and it reduced after INU administration (Figure 2G). In addition, cluster analysis showed that a clear differentiation was observed at the genus level among the Chow, HFD, and INU groups (Figure 2H).

**Figure 3:**
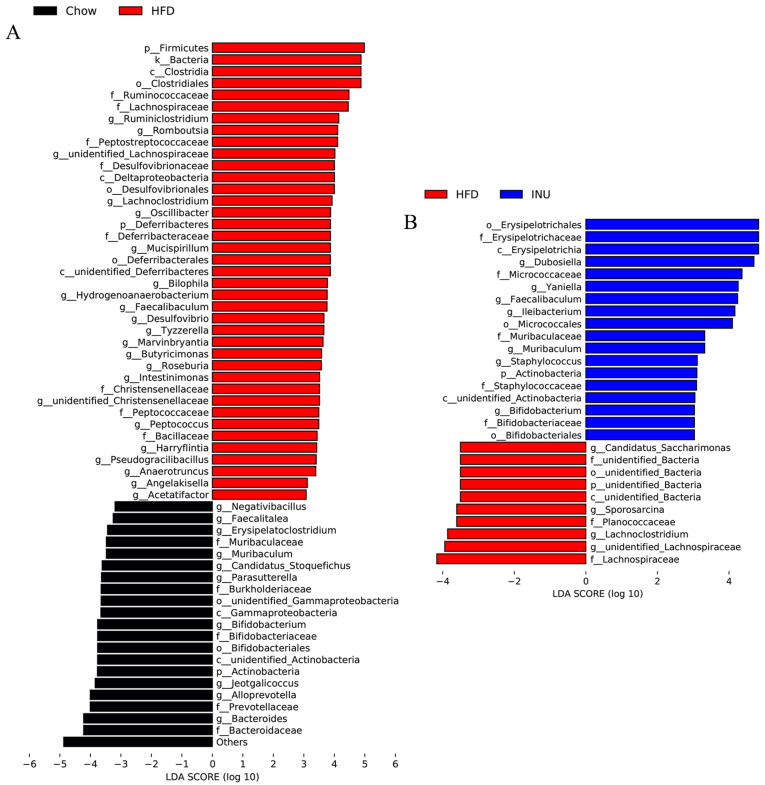
Inulin alters the gut microbiota at the genus level in high-fat diet (HFD)-induced obese mice. Linear discriminant analysis effect size (LEfSe) of the gut microbiota and the corresponding LDA score values (**A**) between mice receiving normal chow (Chow) and HFD (HFD) and (**B**) between mice receiving HFD (HFD) and HFD supplemented with inulin (INU).

**Figure 4:**
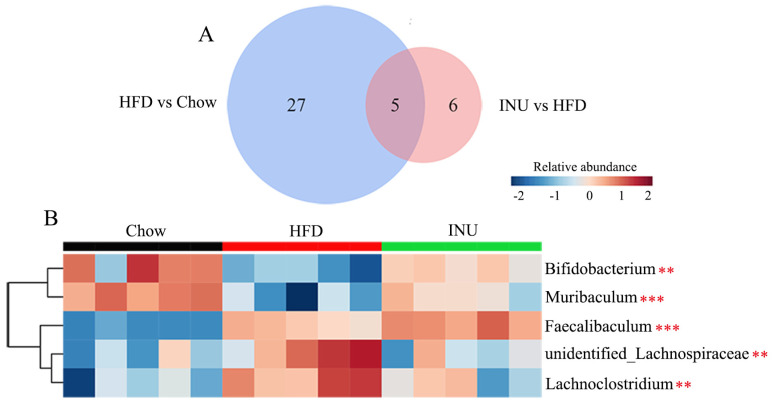
Inulin modifies the gut microbiota at the genus level in high-fat diet (HFD)-induced obese mice. (**A**) The Venn diagram of the gut microbiota identified from the LEfSe; (**B**) heatmap showing changes in 5 common key gut microbes identified from the Venn diagram in mice receiving normal chow (Chow), HFD, and HFD supplemented with inulin (INU). The significance of the differences among the three groups was assessed via one-way ANOVA. Significant level: ** *p* < 0.01; *** *p* < 0.001.

Next, the differential gut bacteria between the Chow and HFD groups (Figure 3A) and between the HFD and INU groups (Figure 3B) were identified by linear discriminant analysis effect size (LEfSe). The Venn diagram was used to select the specific gut microbes at the genus level that were significantly disrupted by HFD and also markedly altered after INU treatment;five gut bacteria were identified (Figure 4A) and presented as a heatmap in Figure 4B. We observed that the relative abundances of *Bifidobacterium* and *Muribaculum* were notably decreased in the HFD-induced obese mice compared with those of the chow-fed mice, while their abundances were significantly increased in the obese mice after INU treatment (Figure 4B). The HFD-induced obese mice had notably higher abundances of *unidentified_Lachnospiraceae* and *Lachnoclostridium* than the chow-fed mice, which could be suppressed after INU intake. Additionally, the relative abundance of *Faecalibaculum* was significantly higher in the HFD-induced obese mice than in the chow-fed mice and further increased after INU treatment, as shown in Figure 4B. Thus, our results suggest that inulin can partially reshape the gut microbiota in HFD-induced obese mice.

### 2.3. The Protective Effect of Inulin on Obesity-Related Disorders Is Transferable by FMT

**Figure 5:**
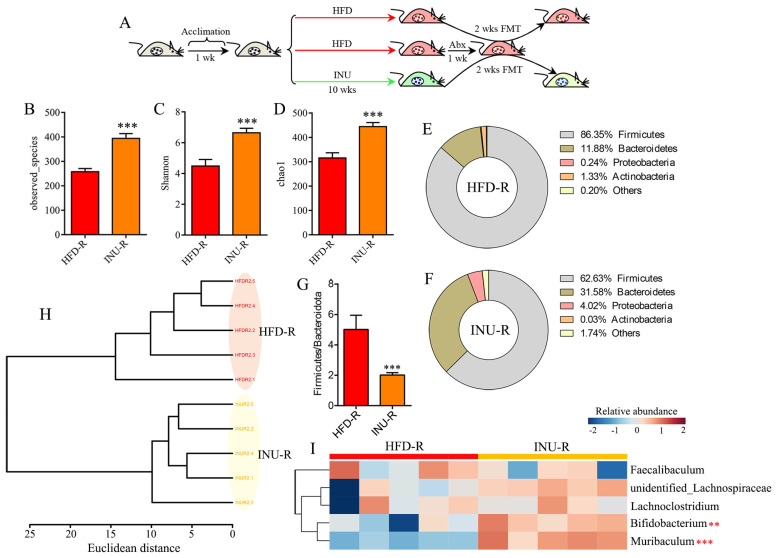
Fecal microbiota transplantation (FMT) from inulin-dosed mice shapes the gut microbiota in high-fat diet (HFD)-induced obese mice. (**A**) FMT process: fecal microbiota either from inulin (INU)-treated or vehicle-treated obese donor mice were transferred to obese recipient mice (INU-R and HFD-R) for 2 weeks; (**B**)observed species, (**C**) Shannon, and (**D**) chao1 indexes of the gut microbiota in INU-R and HFD-R mice; (E, F) the percentages of the gut microbiota at the phylum level in (**E**) HFD-R and (**F**) INU-R mice; (**G**) the ratio of *Firmicutes*/*Bacteroidetes*; (**H**) cluster analysis based on Ward’s method with Euclidean distance using the gut microbiome at the genus level; (**I**) heatmap showing changes in 5 common key gut microbes identified from the LEfSe and Venn diagram in Figure 4. The significance of the differences between two groups was evaluated by two-tailed unpaired Student’s *t*-test. The significance of the differences among the three groups was assessed via one-way ANOVA. Significant level: ** *p* < 0.01; *** *p* < 0.001.

To verify whether the gut microbiota is a causal factor for the advantageous effect of inulin on obesity-related disorders, HFD-induced obese mice were subjected to a 2-week fecal microbiota transfer from either INU-treated or vehicle-treated obese donor mice (INU-R and HFD-R), as shown in Figure 5A. Our results demonstrate that the INU-R mice had a significant increase in the gut microbial α-diversity, including the observed species (Figure 5B), Shannon (Figure 5C), and chao1 (Figure 5D), compared with that of the HFD-R mice. Compared with the HFD-R mice, the percentages of *Firmicutes* and *Actinobacteria* were reduced and the percentages of *Bacteroidetes* and *Proteobacteria* were increased in the INU-R mice (Figure 5E,F). Moreover, the ratio of *Firmicutes*/*Bacteroidetes* was drastically lower in the INU-R mice than in the HFD-R mice (Figure 5G). At the genus level, the cluster analysis shows a differentiated microbial pattern between the INU-R and HFD-R mice (Figure 5H). Figure 5I illustrates the changes in the relative abundances of key gut microbes that were significantly regulated by INU treatment in the intervention study, where we found that INU-R mice had significantly higher abundances of beneficial bacteria, including *Bifidobacterium* and *Muribaculum*.

**Figure 6:**
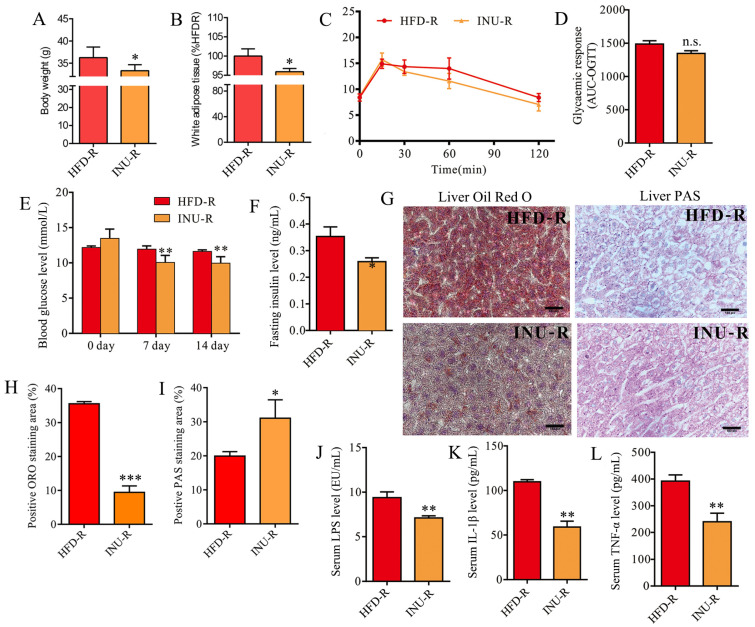
Fecal microbiota transplantation (FMT) from inulin-dosed mice alleviates obesity-related disorders in high-fat diet (HFD)-induced obese mice. (**A**) Body weight; (**B**) white adipose tissue weight; (**C**) oral glucose tolerance test (OGTT); (**D**) area under the curve (AUC) of the OGTT; (**E**) fasting glucose level; (**F**) fasting insulin level; (**G**) histopathological examination: the hepatic sections were stained with oil red O and periodic acid–Schiff (PAS) for evaluating lipid droplets and glycogen, respectively; scale bar =200 μm; (**H**) the percentage of positive ORO staining area; (**I**) the percentage of positive PAS staining area; (**J**) serum lipopolysaccharide (LPS) level; (**K**) serum IL-1β level; (**L**) serum TNF-α level. The significance of the differences between the two groups was evaluated by two-tailed unpaired Student’s *t*-test. Group: HFD-R, HFD-induced obese recipient mice receiving the fecal microbiota from HFD-fed donor mice; INU-R, HFD-induced obese recipient mice receiving the fecal microbiota from inulin-treated HFD-fed donor mice. Significant level: * *p* < 0.05; ** *p* < 0.01; *** *p* < 0.001; n.s., no significant difference.

After a 2-week FMT period, body weight (Figure 6A) and WAT accumulation (Figure 6B) were significantly decreased in the INU-R mice relative to the HFD-R mice. According to the OGTT results, we found that the glucose clearance ability was slightly but not significantly improved in the INU-R mice relative to the HFD-R mice (Figure 6C,D). The INU-R mice possessed significantly reduced levels of fasting blood glucose and fasting insulin compared with the HFD-R mice, as shown in Figure 6E,F, respectively. Figure 6G–I illustrate that reduced lipid droplet and increased glycogen synthesis were observed in the livers of the INU-R mice compared with the HFD-R mice. Additionally, relative to the HFD-R mice, significantly lower levels of LPS (Figure 6J), IL-1β (Figure 6K), and TNF-α (Figure 6L) were detected in the serum of the INU-R mice. Therefore, these results suggest that inulin-reconstructed gut microbiota alleviated obesity and related disorders in HFD-fed mice.

## 3. Materials and Methods

### 3.1. Animal

Male C57BL/6 mice (body weight = 20 ± 2.0 g; age = six weeks) were obtained from the Charles River (Beijing, China) and kept in a specific pathogen-free (SPF) facility at the Laboratory Animal Center of Wenzhou Medical University (WMU, Wenzhou, China). The mice were housed under controlled conditions, including the temperature of 22.0 ± 1.0 °C, humidity of 55.0 ± 5.0%, and light/dark cycle of 12/12 h. During the experiment, all the animals had unlimited access to mice chow and water, both of which were sterilized via irradiation and steam, respectively. The animal care and procedure were performed according to the Guide for the Care and Use of Laboratory Animals and obtained ethical approval from the Institutional Animal Care and Use Committee of WMU (ID: xmsq2020-0502).

### 3.2. Inulin Preparation and Treatment

Inulin derived from Dahlia tubers was obtained from Aladdin, China (CAS number: 9005-80-5) and consisted of one glucose residue and about 36 fructose residues linked by β-(2,1) glycosidic bonds, as shown in Figure 1A. After acclimatization for one week, the mice were fed with a normal chow diet (Chow, n = 11; AIN93M: 10% kcal from fat, 76% kcal from carbohydrate, and 14% kcal from protein; total calories: 3601 kcal/kg); a high-fat diet (HFD, n = 11; D12492: 60% kcal from fat, 20% kcal from carbohydrate and 20% kcal from protein; total calories: 5160 kcal/kg); or HFD supplemented with inulin (INU, n = 11) at a dose of 1.0 g/kg body weight by oral gavage at 8:30 every morning for 10 weeks (Figure 1B). Inulin solution was prepared in sterile water at a concentration of 0.20 g/mL. During the experiment, three mice were housed in each cage and given food in three separate cups. The leftover chow was weighed at 8:30 every morning using an electronic balance. The daily calorie intake was calculated by subtracting from the total delivered food, multiplying by total calories, and then dividing by the number of mice. Three cages were assessed in this study.

### 3.3. Fecal Microbiota Transplantation

In this study, fecal microbiota transplants from inulin-dosed or HFD-fed donor mice to HFD-induced obese recipient mice were carried out in line with a previous method, with a slight modification [17]. The indigenous intestinal microbiota of the obese mice was depleted by the administration of an antibiotic cocktail containing ciprofloxacin of 0.2 g/L, vancomycin of 0.5 g/L, and metronidazole of 1 g/L in their drinking water for 3 consecutivedays. Fresh fecal samples were obtained from the donor mice, and bacterial suspension was prepared through resuspending the fecal sample in sterile phosphate-buffered saline (PBS) at a ratio of 1:20 (*w*/*v*). The mixture was then subjected to avortex for 1 min and a centrifuge at 4 °C for 5 minat 1000 g. Finally, the bacterial suspension (200 μL) was transferred to the HFD-induced obese mice once a day via intragastric administration for 2 weeks.

### 3.4. Oral Glucose Tolerance Test

In this study, the oral glucose tolerance test (OGTT) was employed to assess the glucose clearance capacity of the mice. In brief, the mice were fasted for 12h overnight, after which glucose solution was administered orally via gavage at a dose of 1.0 g/kg. Subsequently, the level of blood glucosewas determined by a tail sample at 0, 15, 30, 60, and 120 min intervals with a handheld glucometer (ACCU-CHEK Active, Mannheim, Germany). Moreover, the fasting insulin level was also measured using the insulin assay kit according to the manufacturer’s instructions (Nanjing Jiancheng Bioengineering Institute, Nanjing, China).

### 3.5. Sample Collection

In this study, fresh fecal pellets were collected from the metabolic cage prior to sacrifice. The mice were weighed and then anesthetized using isoflurane, and a blood sample was obtained by cardiac puncture. The blood sample was centrifuged at 3000× *g* for 15 min at 4 °C to separate the serum sample. White adipose tissues were obtained from the epididymal region, and liver samples were also carefully separated. All the samples were promptly frozen in liquid nitrogen and stored at −80 °C until use.

### 3.6. 16S rRNA Gene Sequencing and Analysis

Total DNA was extracted from fecal pellets using the TIANamp Stool DNA Kit based on the manufacturer’s protocol (TianGen, Beijing, China). A spin column-based method with a silica membrane was employed to bind the DNA under specific salt and pH conditions. After centrifugation, the sample was subjected to a buffer system with InhibitEX Tablet to remove impurities and acquire high-purity and full-length gDNA extract. The DNA concentration was measured using agarose gel electrophoresis (AGE, 1%). The V3-V4 regions of the bacterial 16S rRNA gene were amplified by the universal primers 806R (5′-GGACTACHVGGGTWTCTAAT-3′) and 515F (5′-GTGCCAGCMGCCGCGGTAA-3′). The PCR product was subsequently subjected to purification bythe QIAquick gel extraction kit (Qiagen, Hilden, Germany) and sequenced using an Illumina HiSeq2500 PE250 sequencer (San Diego, CA, USA) at Novogene (Beijing, China).

The raw FastQ files were subjected to bioinformatics analysis. Firstly, the raw tags were filtered and merged into the clean tag using QIIME (v1.7.0) software. Subsequently, the UCHIME algorithm (v7.0.1001) was used to detect the effective tag, and operational taxonomic units (OTUs) were produced by clustering non-chimera clean tags with a 97% similarity threshold under the UPARSE pipeline (v7.0.1001). The Mothur method integrated with the SILVA database was utilized to achieve taxonomy annotation with a confidence threshold of 80%. Lastly, the α- and β-diversity of the gut microbiota were analyzed using QIIME software (v1.7.0) and R software (v3.5.3).

### 3.7. Histopathological Examination

In this study, mice were anesthetized using isoflurane and sacrificed via normal saline perfusion. Then, liver tissues (n = 3) were collected, fixed in a 4% paraformaldehyde solution prepared in PBS buffer (0.1 M, pH = 7.5), and subsequently subjected to dehydration via an ethanol gradient series. The tissue samples were embedded in paraffin and sectioned into 5 μm slices with a slicing machine (Leica, Germany). The liver sections were subsequently stained with periodic acid–Schiff (PAS) for evaluating the level of glycogen. In order to assess lipid droplets, liver tissues (n = 3) were collected, frozen, and embedded in optimal cutting temperature compounds. The tissue samples were sectioned into 5 μm slices with a slicing machine and stained with oil red O (ORO). The Nikon ECLIPSE Ti microscope was utilized for image acquisition, and the positive staining area was measured with ImageJ software (NIH, Bethesda, MD, USA).

### 3.8. Serum Lipopolysaccharide and Cytokine Detection

The ELISA kits for measuring serum lipopolysaccharide (LPS) and cytokines were purchased from Nanjing Jiancheng Bioengineering Institute (Nanjing, China). To assess the level of serum LPS, it was first diluted 10-fold and heated at 70 °C for 10 min; this was followed by measurement via an ELISA kit using Limulus amoebocyte extract. The IL-1β and TNF-α levels were also determined by utilizing amouse-specific ELISA kit in line with the manufacturer’s instructions.

### 3.9. Statistical Analysis

All the animals were randomly subjected to the experimental procedures, such as housing, feeding, and FMT, as well as sample collection and analysis. To assess the statisticalsignificance of the differences between the two groups, a two-tailed unpaired Student’s *t*-test was conducted using SPSS 22.0 software (SPSS, Inc., Chicago, IL, USA). The statisticalsignificance of the differences among the three groups was assessed via one-way ANOVA in SPSS 22.0 software. A repeated-measure ANOVA was performed to examine the statistical significance of a variable’s variation trend using SPSS 22.0 software. The statistical significance was defined at a level of *p* < 0.05. Cluster analysis was carried out via Ward’s method with Euclidean distance using R software (v2.15.3).

## 4. Discussion

Inulin has been widely regarded as a prebiotic to improve obesity-related disorders. Weitkunat et al. reported that inulin supplementation reduced weight gain and hepatic steatosis in HFD-fed mice [35]. Li et al. found that medium- and long-chain inulin supplementation could decrease chronic inflammation as well as increase insulin sensitivity in obese mice [30]. In clinical trials, a systematic review conducted by Fernandes et al. which included 534 overweight/obese adults revealed that inulin eating could reduce inflammatory responses, but more randomized controlled trials are required to support this effect for its clinical application [27]. In the current study, we provided more evidence that inulin serves a beneficial role in the prevention of obesity and its associated disorders in HFD-fed mice.

The advantageous effect of inulin on obesity-related disorders has been associated with the modifications of the gut microbiota, as demonstrated in both animal [26,36] and human [28,37] studies. Of particular significance is the discovery that the transfer of fecal microbiota from inulin-treated mice to HFD-induced obese mice via FMT effectively alleviated a series of obesity-associated disorders, confirming the gut microbiota as a crucial target in the anti-obesity effects of inulin. The gut microbiota in both obese humans and animals has been characterized by a higher *Firmicutes-to-Bacteroidetes* (F/B) ratio and lower microbiota diversity [38,39]. Our findings indicate that treatment with inulin resulted in a significant reduction in the F/B ratio and an increase in the gut microbiota diversity in HFD-induced obese mice, which can be successfully transferred by FMT. In addition, we found that inulin supplementation notably increased the relative abundances of *Bifidobacterium* and *Muribaculum* in obese mice induced by HFD, which can also be transmitted to recipient obese mice. *Bifidobacterium* has been widely recognized as a probiotic bacterium and possesses health-promoting properties, including anti-obesity [40,41]. A lower abundance of *Bifidobacterium* was detected in obese subjects [42,43]. Moreover, supplementation with *Bifidobacterium* has been revealed to relieve obesity and its associated disorders in both animal [44,45] and clinical [46,47] trials. Notably, some plant polysaccharides have the potential to act as prebiotics and thereby prevent the development of metabolic syndrome by enriching the proportion of *Bifidobacterium* in HFD-induced obese mice [48,49]. Dewulf et al. reported that treatment with inulin-type fructans significantly increased *Bifidobacterium* and reduced serum LPS level in obese women [50]. Additionally, the genus *Muribaculum* was also reported to be negatively linked with obesity phenotypes [51]. In our previous study, we reported that dietary fiber from *Dendrobium officinale* protected against HFD-induced obesity and improved glucose metabolism by altering the gut microbiota, including increased *Muribaculum* in mice [17]. *Muribaculum* has been reported to be a contributor of carbohydrate esterase (CE14) and glycoside hydrolase (GH37) by Xu et al. [52], suggesting that *Muribaculum* may have the potential to use inulin. However, whether the *Muribaculum* genus contributes to the mediation of the beneficial role of inulin or represents a potential probiotic candidate for obesity prevention still needs to be further explored in both basic and clinical studies. Relative to *Bifidobacterium*, less information is presently available concerning the role of *Muribaculum* in the onset and progress of obesity; so, whether the *Muribaculum* genus represents a potential probiotic candidate for obesity prevention still needs to be further explored in both basic and clinical studies. In this study, our results suggest that the genera *Bifidobacterium* and *Muribaculum* may play an important role in the favorable effect of inulin on obesity-associated disorders.

## 5. Conclusions

In this study, we reported that treatment with inulin alleviated body weight gain and fat accumulation as well as improved glucose metabolism and inflammation in HFD-induced obese mice. These protective effects of inulin could be partially transferable by FMT, and *Bifidobacterium* and *Muribaculum* were identified as key bacterial genera. Our results confirmed that the gut microbiota serves as a crucial target in the anti-obesity effects of inulin. In the future work, these findings can be confirmed in the human intervention trials to achieve its translational applications.

## Data Availability

The data presented in this study are openly available in FigShare at https://doi.org/10.6084/m9.figshare.22215133.v1.
